# Effect of subclavian vein diameter combined with perioperative fluid therapy on preventing post-induction hypotension in patients with ASA status I or II

**DOI:** 10.1186/s12871-024-02514-9

**Published:** 2024-04-10

**Authors:** Bin Wang, Kangli Hui, Jingwei Xiong, Chongya Yang, Xinyu Cao, Guangli Zhu, Yang Ang, Manlin Duan

**Affiliations:** 1 Department of Anesthesiology, Jinling College affiliated to Nanjing Medical University, Zhongshan East Road #305, Nanjing, Jiangsu Province 210002 China; 2 College of Anesthesiology, Xuzhou Medical University, Xuzhou, Jiangsu 221004 China; 3 Department of Anesthesiology, Affiliated Jinling Hospital, Medical School, Nanjing University, Nanjing, Jiangsu Province 210002 China; 4 Department of Anesthesiology, Nanjing BenQ Medical Center, The Affiliated BenQ Hospital of Nanjing Medical University, Nanjing, Jiangsu 210019 China

**Keywords:** Induction of general anesthesia, Post-induction hypotension, Subclavian vein collapsibility index, Subclavian vein variability, Perioperative fluid therapy

## Abstract

**Background:**

Perioperative hypotension is frequently observed following the initiation of general anesthesia administration, often associated with adverse outcomes. This study assessed the effect of subclavian vein (SCV) diameter combined with perioperative fluid therapy on preventing post-induction hypotension (PIH) in patients with lower ASA status.

**Methods:**

This two-part study included patients aged 18 to 65 years, classified as ASA physical status I or II, and scheduled for elective surgery. The first part (Part I) included 146 adult patients, where maximum SCV diameter (dSCV_max_), minimum SCV diameter (dSCV_min_), SCV collapsibility index (SCV_CI_) and SCV variability (SCV_variability_) assessed using ultrasound. PIH was determined by reduction in mean arterial pressure (MAP) exceeding 30% from baseline measurement or any instance of MAP < falling below 65 mmHg for ≥ a duration of at least 1 min during the period from induction to 10 min after intubation. Receiver Operating Characteristic (ROC) curve analysis was employed to determine the predictive values of subclavian vein diameter and other relevant parameters. The second part comprised 124 adult patients, where patients with SCV diameter above the optimal cutoff value, as determined in Part I study, received 6 ml/kg of colloid solution within 20 min before induction. The study evaluated the impact of subclavian vein diameter combined with perioperative fluid therapy by comparing the observed incidence of PIH after induction of anesthesia.

**Results:**

The areas under the curves (with 95% confidence intervals) for SCV_CI_ and SCV_variability_ were both 0.819 (0.744–0.893). The optimal cutoff values were determined to be 45.4% and 14.7% (with sensitivity of 76.1% and specificity of 86.7%), respectively. Logistic regression analysis, after adjusting for confounding factors, demonstrated that both SCV_CI_ and SCV_variability_ were significant predictors of PIH. A threshold of 45.4% for SCV_CI_ was chosen as the grouping criterion. The incidence of PIH in patients receiving fluid therapy was significantly lower in the SCV_CI_ ≥ 45.4% group compared to the SCV_CI_ < 45.4% group.

**Conclusions:**

Both SCV_CI_ and SCV_variability_ are noninvasive parameters capable of predicting PIH, and their combination with perioperative fluid therapy can reduce the incidence of PIH.

## Induction

Intraoperative hypotension (IOH) presents a prevalent risk for patients undergoing surgical procedures under general anesthesia. It is associated with postoperative major complications, including renal insufficiency, myocardial injury, and increased mortality rates in non-cardiac surgeries [1–3]. In a previous study, approximately 87% of the subjects experienced one or more hypotensive events [4]. Moreover, post-induction hypotension (PIH) often develops in patients experiencing IOH during general anesthesia, typically manifesting between anesthesia induction and the initiation of surgical stimulation [5]. Hence, preventing PIH in patients undergoing elective surgery is of considerable clinical importance.

Baseline mean arterial pressure (MAP) < 70 mmHg, ASA physical status III and IV, the use of propofol and high fentanyl doses, and being above 50 years old are risk factors for PIH [6]. Additionally, patients with high sympathetic tone, autonomic dysfunction and decreased blood volume due to perioperative fasting and bowel preparation are also susceptible to developing PIH [7–9]. A study reported that 59.0% of patients who developed PIH might have hypovolemia before anesthesia induction [10]. Previous studies have indicated that inadequate volume before anesthesia induction is the primary cause of PIH and perioperative fluid therapy in surgical patients before induction reduces the incidence of PIH while promoting more stable intraoperative circulation [11, 12]. Resting pupil size and maximum constriction velocity, as well as heart rate (HR) variability can predict PIH, but these predictive indices do not comprehensively assess preoperative blood volume status in its development [13–15]. Ultrasound diagnostic techniques, as noninvasive procedures, are becoming increasingly popular for assessing intravascular volume status [16].

Ultrasound measurement of inferior vena cava (IVC) diameters has been proposed as a reliable predictor of hypotension following general anesthesia induction in patients with spontaneous respiration [17]. However, the use of IVC measurements might not be suitable for assessing patients with conditions such as high intra-abdominal pressure, abdominal wounds, pneumoperitoneum, extensive subcutaneous emphysema, and morbid obesity [18]. Thus, we opted for the subclavian vein (SCV) over the IVC due to the SCV’s superficial location and its coverage by the clavicle, which can reduce the compression of the ultrasound probe. And a preceding investigation noted a satisfactory correlation between the intravascular volume status of the SCV and IVC [19].

Based on the above-mentioned research findings, we assessed the inner diameter of the SCV and other pertinent parameters to determine their predictive potential for PIH in patients with lower ASA physical status (I or II) in the first part of our study (Part I). In addition, we explored the utility of the SCV diameter and other relevant parameters, in conjunction with fluid therapy, for mitigating PIH in the second part of our study (Part II).

## Materials and methods

### Patients

This prospective study received ethical approval from the Ethics Committee of Jinling Hospital, a prominent Chinese tertiary-level teaching hospital, on September 29, 2022 (Approval No: 2022DZKY-084-01). The study was duly registered with the Chinese Clinical Trial Registry under the registration number ChiCTR2300068562 (registration date: 23/02/2023). Prior to participation, all eligible patients provided comprehensive written informed consent. Inclusion criteria were: Individuals aged between 18 and 65 years, classified as ASA physical status I or II, and scheduled for elective surgery under general anesthesia. Patients were excluded if they had a medical history of hypertension, diabetes mellitus, acute kidney injury, coagulation dysfunction, implanted pacemaker/cardioverter devices, tricuspid failure, right-sided heart disease, portal hypertension, obstructive lung diseases, had taken angiotensin-converting enzyme inhibitors or angiotensin receptor blockers; or were undergoing procedures in lateral or prone positions. Additionally, patients with incomplete data (pertaining to SCV, MAP, or HR) during the study period were excluded from the trial.

### Subclavian vein ultrasonography

**Fig. 1:**
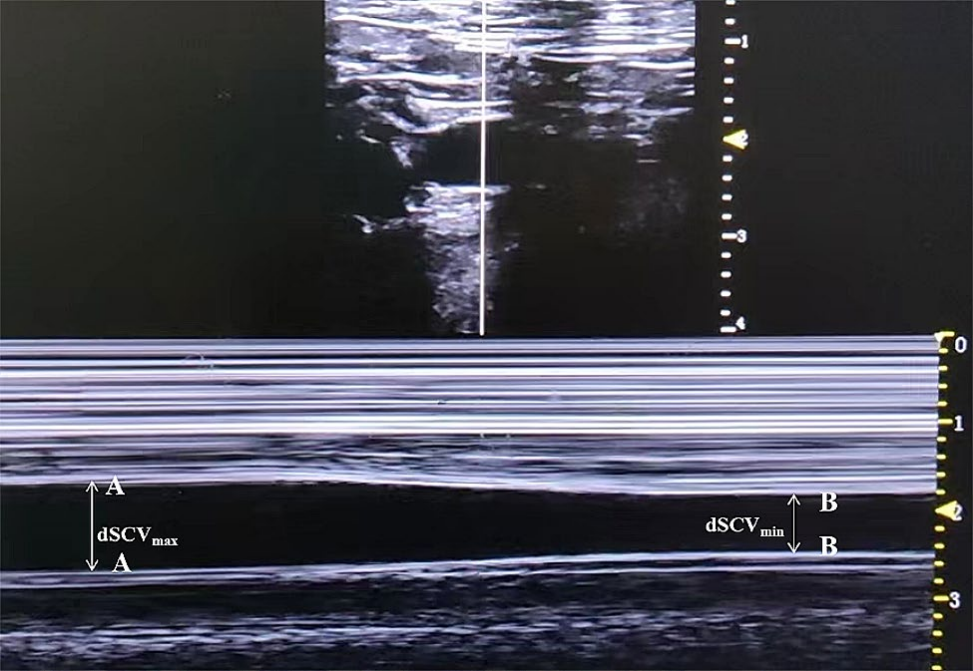
M-mode ultrasonography of the subclavian vein

Before the SCV examination, all patients would wait in the anesthetic preparation room for ≥ 5 min prior to entering the operating room and were conscious, laying supine, and spontaneously breathing. A 4–15 MHz linear probe of an ultrasound unit (Wisonic, China) was used to perform ultrasound measurements of the right SCV diameter in all patients. To obtain the best view of the SCV during the study, the probe was placed beneath the proximal part of the middle of the clavicle and the area below the clavicle was scanned. Patients were directed to perform a deep, intentional inhalation, followed by a gradual and relaxed exhaling process. After locating the target vein, the change in dynamic diameter over time was recorded using M-mode imaging, which was subsequently used to identify and measure the minimum (dSCV_min_) and maximum (dSCV_max_) venous dimensions over the respiratory cycle. The SCV Collapsibility Index (SCV_CI_) and SCV variability (SCV_variability_) were calculated using the following formulas; SCV_CI_ = (dSCV_max_ – dSCV_min_)/dSCV_max_ * 100%, and SCV_variability_ = (dSCV_max_ – dSCV_min_)/ (dSCV_max_ + dSCV_min_)/2 * 100% [20, 21]. The mean of three measurements was selected. All these measurements were obtained by one anaesthesiologist with extensive sonography experience (Fig. 1).

### Anesthesia management

All patients were fasted for 12 h, and none had been pre-medicated before surgery. All patients’ vital signs (pulse oxygen saturation, respiratory rate, blood pressure, and electrocardiogram) were monitored. The anesthesia induction regimen followed a standard conventional sequence induction: 0.04 mg/kg midazolam + 0.3 µg/kg sufentanil + 2 mg/kg propofol + 0.15 mg/kg cisatracurium. Using a video laryngoscope, an experienced anesthesiologist performed tracheal intubation 3 min after administering the muscle relaxant. Subsequently, mechanical ventilation was administered using a volume of 8 ml/kg (based on ideal body weight), accompanied by a fresh gas flow rate of 2 L/min. In parallel, a Ringer’s acetate solution was consistently infused at a rate of 10 ml/kg/h for all patients participating in the study.

### Blood pressure measurements

Invasive blood pressure monitoring was performed in all patients using a 20-gauge arterial catheter (Supercath Ztu-V, Japan) inserted into the radial artery following local lidocaine infiltration before induction. The catheter was then connected to a pressure sensor (Hisern, Zhejiang) flushed with heparinized saline. After adjusting the zero pressure, MAP was subsequently recorded every 1 min by the monitor (Mindray, China).

### Sample size calculations and data collection

In Part I study, the sample size was determined using following formula: N = [(Z_α/2_ + Z_β_) S/δ]^2^, and the standard deviation was obtained based on the results of previous studies [22], δ = (0.25–0.5) S. The required number of cases was calculated to be 126 (α = 0.05, power = 80%). A sample size of 140 (assuming a 10% dropout rate) was enrolled to achieve sufficient statistical power. Demographic data (comorbid diseases, weight, height, sex, and age) were obtained. Moreover, HR and MAP were measured every minute until 10 min after intubation, with baseline MAP defined as the blood pressure value 1 min before induction. Episodes of PIH were defined as a > 30% decrease in MAP from the baseline level or any recorded period of MAP < 65 mmHg for ≥ 1 min between induction and 10 min post-intubation. Patients were treated with intravenous boluses of phenylephrine (20 µg) if MAP was < 65 mmHg or if it decreased by > 30% from the baseline level and lasted for ≥ 1 min. Bradycardia patients (HR < 50 beats/min) were treated with atropine (0.5 mg). Based on the presence or absence of hypotension during the study, patients were classified into two groups: PIH and Non-PIH. A different anesthesiologist collected and compiled all the data.

For the Part II study, we utilized PASS 15.0 software to estimate the sample size based on the observed incidence of PIH of 48.6% from our Part I study. Therefore, we assumed the significant difference α = 0.05, power of a test β = 0.8, and accounted for a 10% dropout rate. The sample size was determined to be 129. Patients were stratified into group L (SCV_CI_ ≥ 45.4%) and group H (SCV_CI_ < 45.4%) based on their pre-rehydration SCV_CI_ values. Patients in group L received an intravenous bolus of colloid solution at a volume of 6 ml/kg over 20 min before induction [11]. Conversely, patients in group H did not receive additional fluid therapy before induction. dSCV_max_, dSCV_min_, SCV_CI_, SCV_variability_, MAP and HR were recorded before and after rehydration. MAP and HR were assessed every minute until 10 min post-intubation, with the minimum value recorded. Furthermore, the 146 patients from Part I study were categorized into two groups: group L_1_ (SCV_CI_ ≥ 45.4%) and group H_1_ (SCV_CI_ < 45.4%). Subsequently, the incidence of PIH was compared among the four groups.

### Statistical analysis

Data collected were compiled using Microsoft Excel (v 2304, Microsoft, USA). The Kolmogorov-Smirnov test was used to assess the normality of the collected data with normally distributed results reported as mean ± standard deviation (‾x ± s), and inter-group differences compared using an independent sample t-test. Non-normally distributed data were expressed as medians (interquartile ranges), and the Mann-Whitney U test was used to compare the differences. For categorical variables, analysis was conducted using the chi-square test, presenting results in numerical values and percentages.

A binary logistic regression analysis was utilized to investigate the association between SCV parameters and the occurrence of PIH. Based on clinical practice and previous PIH research, confounding variables selected for this study included ASA physical status, sex, age, body mass index (BMI), albumin levels, baseline MAP and baseline HR [1, 6, 23]. Based on the results of these analyses, the receiver operating characteristic (ROC) was performed to determine the ability of SCV parameters to predict PIH for all patients. The calculation of the area under the curve (AUC), optimal threshold values, and a 95% confidence interval (CI) was also executed. All statistical computations were carried out using SPSS version 25.0 (IBM, USA). Results displaying a significance level of *P* < 0.05 were deemed statistically noteworthy.

## Results

### Part I: to investigate the predictive value of SCV diameter parameters for PIH in ASA I or II patients undergoing elective surgery

**Fig. 2:**
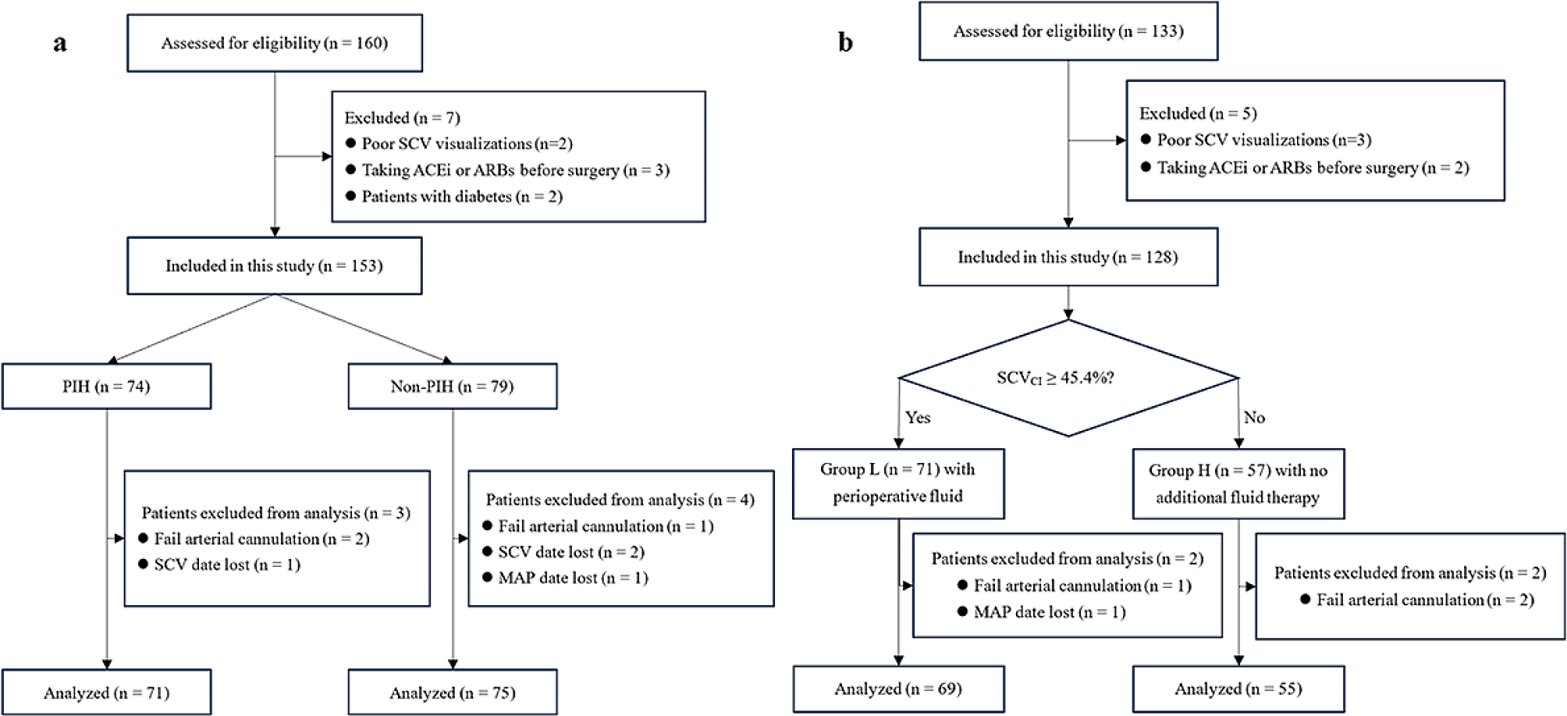
(**a**) Study flow chart of Part I; (**b**) Study flow chart of Part II

A total of 146 patients were included in the final analysis for Part I study (Fig. 2a). According to the study criteria, 71 out of 146 patients (48.6%) developed hypotension following general anesthesia induction. Notably, there were no differences in sex, BMI, ASA physical status, red blood cell count and hemoglobin between the PIH and Non-PIH groups (*P* > 0.05). Patients who developed PIH were older (*P* = 0.002) and had lower hematocrit (*P* = 0.048) and albumin levels (*P* = 0.001) (Table 1). Table 2 revealed that patients who developed PIH had a lower dSCV_max_ (*P* = 0.022) and dSCV_min_ (*P* < 0.001) and a higher SCV_CI_ (*P* < 0.001) and SCV_variability_ (*P* < 0.001).

**Table 1:** Patient baseline characteristics in Part I

	PIH (*n* = 71)	Non-PIH (*n* = 75)	P Value
Age (years)	44.0 ± 12.9	37.3 ± 12.9	0.002
Sex (male/female)	32/39	43/32	0.138
Height, cm	165.0 [160.0-173.0]	168.0 [162.0-173.0]	0.261
BMI, kg/m^2	23.3 ± 2.9	23.7 ± 3.4	0.440
ASA (I/II)	27/44	38/37	0.125
Red blood cell, 10^12/L	4.53 ± 0.57	4.69 ± 0.62	0.102
Hemoglobin, g/L	135.0 [124.0-149.0]	143.0 [130.0-151.0]	0.096
Hematocrit (%)	41.0 [37.7–44.5]	42.9 [39.1–45.7]	0.048
Albumin, g/L	41.8 ± 4.3	44.2 ± 4.4	0.001

BMI, body mass index; ASA, American Society of Anesthesiologists physical status. Normally distributed results were reported as mean ± standard deviation (‾x ± s), while non-normally distributed data were expressed as medians [interquartile ranges]

**Table 2:** Hemodynamic and subclavian vein ultrasound data

	PIH (*n* = 71)	Non-PIH (*n* = 75)	P Value
Baseline MAP, mmHg	93.2 ± 11.7	94.8 ± 9.6	0.353
Baseline HR, beats/min	70.0 [63.0–81.0]	72.0 [60.0–76.0]	0.372
Decrease in MAP (%)	31.5 [24.1–34.9]	18.2 [13.8–22.1]	< 0.001
Percentage change in HR (%)	11.9 [5.3–20.9]	11.3 [5.4–20.5]	0.868
Lowest MAP, mmHg	64.0 [62.0–68.0]	76.0 [73.0–82.0]	< 0.001
dSCV_max_; cm	0.84 ± 0.15	0.89 ± 0.14	0.022
dSCV_min_; cm	0.37 [0.43–0.50]	0.48 [0.56–0.66]	< 0.001
SCV_CI_ (%)	49.4 [45.4–51.3]	37.3 [29.2–43.0]	< 0.001
SCV_variability_ (%)	16.4 [14.7–17.2]	11.5 [8.6–13.7]	< 0.001

MAP, mean arterial pressure; HR, heart rate; dSCV_max_, maximum value of subclavian vein diameter; dSCV_min_, minimum value of subclavian vein diameter; SCV_CI_, subclavian vein collapsibility index; SCV_variability_, subclavian vein variability;$$Decrease\,in\,MAP\, = \,\left( {\frac{{Baseline\,MAP - Minimum\,MAP}}{{Baseline\,MAP}}} \right) \times 100\%$$$$Percentage\,change\,in\,HR = \left( {\frac{{Baseline\,HR - HR\,at\,minimum\,MAP}}{{Baseline\,HR}}} \right) \times 100\%$$

Normally distributed results were reported as mean ± standard deviation (‾x ± s), while non-normally distributed data were expressed as medians [interquartile ranges]

**Fig. 3:**
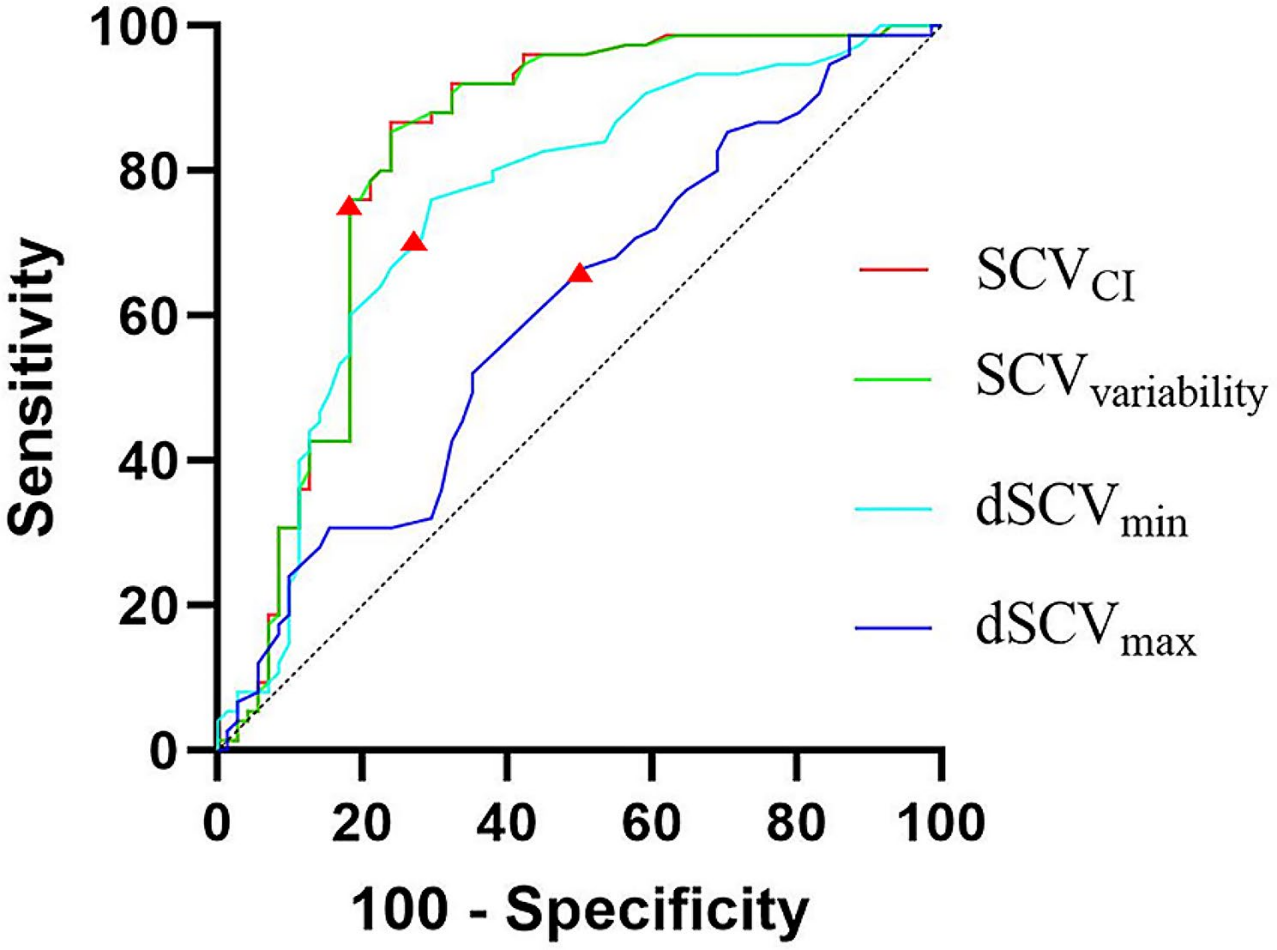
Comparison of Receiver Operating Characteristic (ROC) curves of subclavian vein (SCV) collapsibility index (SCV_CI_), SCV variability (SCV_variability_), and the minimum (dSCV_min_) and maximum (dSCV_max_) SCV dimensions to predict PIH. The triangles on the curves show the optimal cutoff values determined by maximizing the Youden index

The diagnostic accuracy of the ROC curve analysis for predicting PIH was high, with the SCV_variability_ line almost completely overlapping the SCV_CI_ line (Fig. 3). Both SCV_CI_ and SCV_variability_ exhibited AUCs of 0.819 (*P* < 0.001; 95% CI: 0.744–0.893), with optimal cutoff values of 45.4% and 14.7%, respectively. The sensitivity and specificity values for SCV_CI_ and SCV_variability_ were 76.1% and 86.7%, respectively. The AUC for dSCV_min_ was 0.752 (*P* < 0.001; 95% CI: 0.671–0.834), with an optimal cutoff value of 0.48 cm. The sensitivity and specificity values were 70.4% and 76.0%, respectively. The AUC for dSCV_max_ was 0.603 (*P* = 0.031; 95% CI: 0.512–0.695), which was lower than that of dSCV_min_ (0.752). The optimal cutoff value for dSCV_max_ was 0.90 cm, with sensitivity and specificity values of 64.8% of 52.0%, respectively.

Univariate analysis revealed that PIH was related to older age, lower albumin levels, smaller maximum and minimum SCV diameters during deep inhalation, and higher SCV_CI_ and SCV_variability_. Due to the strong collinearity between SCV_CI_ and SCV_variability_, as well as between dSCV_max_ and dSCV_min_, two separate models were employed for the analysis. After adjusting for age, sex, BMI, ASA physical status, albumin, baseline MAP and HR, SCV_CI_ (*P* < 0.001) and SCV_variability_ (*P* = 0.001) were found to be independent PIH predictors (Table 3).

**Table 3:** Multivariate logistic regression

	Unadjusted analysis	Adjusted analysis OR [95% CI]	
	OR [95% CI]	Model 1	Model 2
Age(years)	1.040 (1.014–1.067) ^++^	1.031 (0.982–1.082)	1.029 (0.980–1.081)
Sex			
Male	1.00	1.00	1.00
Female	1.638 (0.851–3.150)	1.081 (0.422–2.767)	1.088 (0.420–2.814)
BMI, kg/m^2	0.960 (0.865–1.065)	0.962 (0.838–1.104)	0.951 (0.827–1.094)
ASA			
ASA I	1.00	1.00	1.00
ASA II	1.674 (0.866–3.236)	1.054 (0.334–3.324)	1.076 (0.336–3.451)
Albumin, g/L	0.879 (0.811–0.952) ^++^	0.875 (0.795–0.963) ^++^	0.873 (0.792–0.962) ^++^
Baseline MAP, mmHg	0.985 (0.956–1.016)	0.964 (0.922–1.009)	0.966 (0.922–1.011)
Baseline HR, beats/min	1.018 (0.993–1.044)	0.995 (0.959–1.032)	0.994 (0.958–1.032)
dSCV_max_; cm	0.071 (0.007–0.706) ^+^	0.806 (0.042–15.621)	—
dSCV_min_; cm	0.002 (0-0.028) ^+++^	—	2.158 (0.016-290.968)
SCV_CI_ (%)	1.118 (1.071–1.166) ^+++^	1.119 (1.066–1.173) ^+++^	—
SCV_variability_ (%)	1.356 (1.213–1.515) ^+++^	—	1.396 (1.154–1.690) ^++^

^+^*P* < 0.05, ^++^*P* < 0.01, ^+++^*P* < 0.001. MAP, mean arterial pressure; HR, heart rate; BMI, body mass index; ASA, American Society of Anesthesiologists physical status; dSCV_max_, maximum value of subclavian vein diameter; dSCV_min_, minimum value of subclavian vein diameter; SCV_CI_, subclavian vein collapsibility index; SCV_variability_, subclavian vein variability; OR, odds ratio; CI, confidence interval

Model 1: Adjusted for Age, Sex, BMI, ASA physical status, Albumin, Baseline MAP, Baseline HR, dSCV_max_ and SCV_CI_.

Model 2: Adjusted for Age, Sex, BMI, ASA physical status, Albumin, Baseline MAP, Baseline HR, dSCV_min_ and SCV_variability_.

### Part II: to explore the value of SCV_CI_ combined with perioperative fluid therapy in preventing PIH in ASA I or II patients

In Part II study, 124 patients were categorized into two distinct groups based on their SCV_CI_ values: group L (SCV_CI_ ≥ 45.4%, *n* = 69) and group H (SCV_CI_ < 45.4%, *n* = 55) (Fig. 2b). There were no significant differences in age, sex, height, BMI, ASA physical status, baseline MAP and HR between the L and H groups (*P* > 0.05). However, the albumin levels in group L were lower than those in group H (Table 4, *P* = 0.018).

**Table 4:** Patient baseline characteristics in Part II

	Group L (*n* = 69)	Group H (*n* = 55)	P Value
Age (years)	41.8 ± 12.3	40.2 ± 11.7	0.468
Sex (male/female)	38/31	27/28	0.508
Height, cm	167.9 ± 8.0	168.1 ± 8.5	0.924
BMI, kg/m^2	23.4 ± 2.8	23.5 ± 3.3	0.751
ASA (I/II)	22/47	22/33	0.348
Albumin, g/L	42.7 ± 6.5	45.1 ± 4.1	0.018
Baseline MAP, mmHg	91.9 ± 5.6	90.3 ± 5.0	0.106
Baseline HR, beats/min	72.9 ± 9.0	71.2 ± 9.5	0.330

BMI, body mass index; ASA, American Society of Anesthesiologists physical status; MAP, mean arterial pressure; HR, heart rate. Normally distributed results were reported as mean ± standard deviation (‾x ± s)

Following perioperative fluid therapy, the SCV_CI_ in group L significantly decreased post-administration compared to pre-administration levels (Table 5, *P* < 0.001). The incidence of PIH in group L and group H was 42.0% and 29.1%, respectively; however, no significant difference was observed between the two groups (*P* > 0.05).

**Table 5:** Subclavian vein collapsibility index before and after rehydration in group L

	Group	Before rehydration	After rehydration	P Value
SCV_CI_ (%)	L (*n* = 69)	50.5[48.5–52.4]	37.6[34.7–39.2]	< 0.001

SCV_CI_, subclavian vein collapsibility index; group L, patients with SCV_CI_ ≥ 45.4% in part II

The 146 patients included in Part I study were divided into group L_1_ (SCV_CI_ ≥ 45.4%) and group H_1_ (SCV_CI_ < 45.4%) based on preinduction SCV_CI_ values to examine the influence of perioperative fluid therapy on preventing PIH. We found that the incidence of PIH in group H and group H_1_ was 29.1% and 21.7%, respectively, and there was no statistically significant difference in the incidence of PIH between the two groups. (*P* > 0.05). Conversely, patients in group L who received perioperative fluid therapy had a significantly lower incidence of PIH compared to group L_1_ (*P* < 0.001).

## Discussion

In this study, we identified that ultrasound measurement of SCV diameter can assist in identifying patients at an elevated risk of developing PIH. Both SCV_CI_ and SCV_variability_ were identified as predictors of PIH during deep inspiration, with an AUC value of 0.819 (0.744–0.893), sensitivity of 76.1%, and specificity of 86.7%. Furthermore, the optimal cutoff values for SCV_CI_ and SCV_variability_ were 45.4% and 14.7%, respectively. An SCV_CI_ ≥ 45.4% before anesthesia induction indicated a significant increase in the risk of post-induction hypotension. Importantly, we found that administering a colloidal solution of 6 ml/kg 20 min before anesthesia induction reduced the incidence of PIH in patients with SCV_CI_ ≥ 45.4%. Therefore, we believe that combining SCV ultrasound with a specific volume of perioperative fluid therapy can effectively reduce the incidence of PIH in patients with ASA I or II.

PIH is a common occurrence encountered by anesthesiologists in clinical activities, primarily attributed to the patient’s hypovolemic state, cardiovascular depression, and the vasodilatory effects of anesthetics [8, 10]. Blood pressure serves as a fundamental indicator reflecting patients’ hemodynamic status. Our study excluded patients who were elderly, had ASA physical status III or IV, were hypertensive and treated with converting enzyme inhibitors, or underwent emergency surgery. We observed a significant drop in blood pressure in the majority of patients following the induction of general anesthesia. This observation may indicate pre-existing hypovolemia prior to anesthesia induction, even after accounting for the effects of anesthetic agents. Thus, it is imperative to evaluate the preoperative intravascular volume status of patients to effectively manage this concern.

Ultrasound measurement of venous diameter offers a noninvasive approach to assessing intravascular volume status [24]. A previous study highlighted the efficacy of the IVC as an indicator for assessing intravascular volume status [25]. However, Kent et al. demonstrated that SCV had a small overall deviation from IVC in collapsibility evaluation and could be superior in velocity measurement; suggesting its potential as a substitute for the IVC to a certain extent [20]. A study reported that SCV_CI_ > 13.4% and SCV_variability_ > 14.3% showed clinical significance in predicting fluid responsiveness, and following a fluid challenge, SCV_CI_ and SCV_variability_ significantly decreased, while dSCV_max_ significantly increased [21]. Choi et al. found that SCV_CI_ during deep inspiration could predict PIH in patients undergoing laparoscopic gallbladder surgery, but no optimal cutoff value for SCV_CI_ was obtained in this study [22]. In Part I study, an optimal cutoff value of SCV_CI_ for predicting PIH was determined to be 45.4%. This difference in SCV_CI_ could be attributed to patients taking deep breaths, resulting in a smaller measurement of dSCV_min_ compared to spontaneous breathing.

The incidence of PIH was 48.6% in Part I study, consistent with rates observed in our hospital (50%) and reported by Zhang et al. (46.7%) [17]. However, the incidence of PIH in our study population differed from that reported by Choi et al. (24.7%) [22]. In our study, we administered anesthetic agents based on patients’ weight to eliminate the influence of anesthetic agents on PIH. The elevated incidence of PIH may be attributed to the 12-hour fasting period, which could induce hypovolemia in patients. In addition, there are several definitions of hypotension. For instance, Bijker et al. showed 140 definitions for IOH [26]. Therefore, various studies use different definitions of hypotension, resulting in varied PIH incidence rates. In this context, hypotension was characterized as either a reduction in MAP exceeding 30% from the initial baseline or a MAP value below 65 mmHg sustained for at least one min. We selected 65 mmHg as the threshold to better ensure patients’ safety and reduce hypotension-induced damage to organs, including the heart, brain, and kidneys [27]. Jor et al. highlighted the presence of diabetes as a PIH risk factor in a study involving 661 patients under general anesthesia [28]. As a result, we excluded patients with diabetes from our study.

In Part I study, older age was associated with a higher risk of PIH; however, it did not emerge as an independent predictor in logistic regression analysis after adjusting for confounding factors. This observation could be attributed to the limited sample size in this study or the exclusion of patients over 65 years of age from the trial. Additionally, we observed that patients with PIH exhibited lower albumin levels compared to those without PIH. Moreover, in Part II study, group L (SCV_CI_ ≥ 45.4%) had lower albumin levels than group H (SCV_CI_ < 45.4%). These observations may be attributed to patients with lower albumin levels having reduced plasma colloid osmotic pressure and water content in plasma.

Perioperative fluid therapy represents an effective strategy for preventing PIH [29]. Moreover, the selection of different types of fluids can yield varying outcomes. A prior investigation indicated that fluid optimization with crystalloids before the induction of general anesthesia did not exert a notable impact on hemodynamic instability [30]. Colloids, such as dextrans, hydroxyethyl starches (HES), gelatins, and albumin, have the advantage of prolonged intravascular retention, and the administration of a small colloid volume before anesthesia induction has been associated with a reduced incidence of PIH [11, 31]. Therefore, HES was selected for perioperative fluid therapy prior to induction in our study. Considering the potential risks associated with HES in patients with coagulation disorders and renal impairment, individuals exhibiting coagulation dysfunction and acute kidney injury were excluded from our study [32, 33]. Further investigation of the data from Part I and Part II revealed a markedly lower incidence of PIH in group L compared to group L_1_, with SCV_CI_ values in group L showing a significant decrease following perioperative fluid therapy. These findings suggest that the perioperative administration of a certain volume of fluid can significantly reduce the incidence of PIH in patients with hypovolemia before induction.

This study exhibited several limitations. Firstly, patients classified as ASA physical status III or higher were excluded from the study due to the potential presence of severe systemic diseases, which could introduce additional risk factors and yield different outcomes. Secondly, our study did not employ techniques such as echocardiography or non-invasive monitoring of cardiac function parameters or cardiac output to directly evaluate systemic volume and cardiac contractility in patients. Moreover, the extended fasting period might have induced hypovolemia, potentially contributing to a higher incidence of PIH. Thirdly, the study was conducted at a single center with a limited sample size in China, possibly introducing biases and limiting the generalizability of the results. Fourthly, the study was not blinded, potentially introducing bias due to anesthesiologists’ awareness of the potential of SCV measurement for assessing fluid responsiveness. Finally, to simulate a clinical setting more closely, the study included patients undergoing various surgical procedures, potentially introducing some degree of variability. Future research should focus on patients undergoing specific types of surgery to investigate the potential role of SCV ultrasound in combination with perioperative fluid therapy under specific conditions, aiming to enhance reliability.

## Conclusions

In conclusion, our study demonstrates that pre-anesthesia ultrasound measurement of SCV diameter can predict PIH to some extent in patients with ASA status I or II. Both SCV_CI_ and SCV_variability_ serve as predictors of PIH. Particularly, in patients with SCV_CI_ ≥ 45.4% before induction of anesthesia, the implementation of perioperative fluid therapy plays a crucial role in significantly reducing the incidence of PIH.

## Data Availability

No datasets were generated or analysed during the current study.

## Abbreviations

IOH: Intraoperative hypotension
PIH: Post-induction hypotension
MAP: Mean arterial pressure
HR: Heart rate
BMI: Body mass index
ASA: American Society of Anesthesiologists physical status
IVC: Inferior vena cava
SCV: Subclavian vein
dSCV_max_: Maximum subclavian vein diameter
dSCV_min_: Minimum subclavian vein diameter
SCV_CI_: Subclavian vein collapsibility index
SCV_variability_: Subclavian vein variability
ROC: Receiver operating characteristic
AUC: Area under the curve
HES: Hydroxyethyl starch
